# Influence of Climate and Diet on Teeth

**Published:** 1897-05

**Authors:** 


ARTICLE V.

### INFLUENCE OF CLIMATE AND DIET ON TEETH.

Read before the Rochester Dental Society, December, 1896.

The subject which was assigned me by your committee to write upon for this evening, is one which I think you will all agree with me, when I say it, is one which requires much study and careful research to accomplish any positive results.

So when a brother came to me at the eleventh hour and asked me to exchange dates with him, I looked at him in despair, but I told him I would do the best I could. What influence has diet on the teeth ? That changes take place in tooth structure resulting from different kinds of food ? I do not propose in this short paper to give tables showing the relative food values; how much bone or how much muscular tissue we can hope to build up from a given quantity of a given kind of food.

All these theories are based for the most part on individual opinions, and are valuable or worthless just in proportion as they are corroborated by other investigation and experiment carried on in a similar manner.

Place a child in an equable climate, give it sufficient nutritious food, let it have pleasant surroundings, allow it to thrive and flourish, then when maturity is reached (providing it had a proper heredity at birth), will it not develop into a perfect, healthy representative of its kind, enjoying perfectly developed teeth ?

The simpler partially mixed diet, if you can comprehend the term, the steady routine diet which goes on from generation to generation, seems to produce the best dentures. The humbler classes of the purer races will show this I think.

The Germans always eat German, and their teeth are better than ours. The Scotchmen always eat Scotch, and their teeth are better than ours. The Americans eat *everything* indiscriminately, and their teeth are poor. I am speaking now of structure, not caries.

The laws governing nutrition are not well enough understood to say that any one kind of diet is an ideal one.

The food must necessarily conform to the climate. Climate possibly has more to do with the proper development of tooth structure than any one kind of diet. If this be the case, then in a climate where sudden extremes of temperature continually prevail, we should expect to find people with a poor quality of teeth; or in an equable climate, better organized tooth structure should be looked for.

This is the exact condition of things we find in our own country. A climate in which the temperature is ever changing from one extreme to the other, accompanied much of the time by a high humidity, and people with teeth of questionable structure.

The people of cold countries, such as laplands, have highly developed teeth, so also have the South Sea Islanders who live in a warm or semi-tropic climate. But these countries are not subject to the sudden extremes of heat and cold that we find in this climate. The people living on the extreme northern end of the Scandinavian peninsula such as Lapps, Finns, etc., are renowned for their strong even dentures, So is the African Negro.

Here again we meet the extremes of climate, the cold and rigorous of the north and the semi-tropic of the south. However, they are both equable.

Now, if we find a people living in a region within the Arctic circle whose chief diet is whale blubber and walrus steak, or possibly reindeer stew, if we find these people with fairly developed dentures; and on the other hand an African living on rice, dates and cocoanuts, who shows a corresponding good set of teeth, then we must look for some other influence than the food they eat for the cause. Nasal catarrh so prevalent in this country is almost unknown in climates of equable temperature. Aside from strumous and syphilitic heredity, nasal catarrh is the result of repeated or long continued exposure to the changes of our American climate. The vaso-motor centres are repeatedly irritated by the sudden thermal changes, until the respiratory portion of the lining membrane of the nasal cavity becomes inflamed and hypertrophied, the disease becomes chronic, and other tracts are involved.

If we find such havoc wrought in the membranes of the nasal air passages, disease so common, so wide-spread, why not look farther and see if tooth development is not influenced by the same climatic condition.

All food assimilation must be governed by the laws of supply and demand.

To convert an element in a food into an element in an organized tissue, there must first be established a demand for that element. Many of the foods which we have to-day are rich in tooth elements, but are utterly worthless as food whereby to supply these elements. Oat meal and milk, that stand-by of so many American breakfast tables, though high in its proportion of lime salts, is I think almost worthless as a tooth builder, owing to its faulty preparation and the manner in which it is eaten, namely, with a spoon, and without the least effort at mastication or mixture with saliva

Among my own people there is a family of four children that have been under my observation since infancy. The elder boy is fourteen years of age. The morning meal has invariably been oat meal and milk since the time they first ate solid food. Their bones and bodies are strong and healthy, but the teeth of the three younger children are almost as poor as the poorest, and I can do nothing with them but rely on pink gutta percha.

Here the diet made strong bones and tough muscles, why did it not make hard teeth ? There was no demand for hard teeth. The oat mush needed no chewing. Phosphate of lime knocked at the door, but found the sign *not wanted.*

Had the oat meal been made into bread, or even eaten dry from the original package, different results might have been obtained. I think that in most cases where the proportion of lime salts to organic matter is found to be below the standard, the cause will be found, not from lack of supply, but from other causes, such as climatic conditions, natural surroundings, or from the one quoted above.

It has been demonstrated beyond question that all *chemical* forms of lime salts are rejected by the system without having been absorbed. According to Lehman and Heiden phosphate of lime added to food is execreted in its entirety without any absorption.

According to Beaumetz phosphate of lime and phosphoric acid are precipitated immediately on reaching the stomach, and are rendered insoluble. Hence the absolute uselessness of prescribing chemical food either to growing children or pregnant women.

Phosphate of lime we must have, but only such forms as are presented to the system in organized combination, will it take up. Some kinds of food have a direct local influence on the teeth, namely, fruits. Oranges, lemons, apples, grapes and berries contain beside sugar and flavoring material, an acid, which by long continued application is capable of dissolving out the lime salts of the teeth. Citric acid, which exists in large proportions in lemon and orange juices, seems to have the strongest corrosive action on the enamel of the teeth of any of the fruit acids.

Tartaric acid or the acid found in grapes comes next in order.

Some tests were made with freshly extracted teeth to show this action of fruit acid. The juice was squeezed or pressed from fresh ripe fruit, the teeth placed in it and allowed to remain for twenty-four hours.

These experiments have been frequently repeated, and no claim is made for originality. To those who have never seen the action of the different fruit acids on enamel, they will prove interesting. I now hope to hear of some more influences of diet on teeth.

### DISCUSSION.

Dr. Sibley : So far as effect of the climate upon the teeth is concerned, I am inclined to believe that, with those people who live in the far North and those who live in the semi-tropics, the diet plays a more important part in the make-up of the teeth than the climate.

These people are in the habit of living upon practically the same form of diet, and digestion goes on uninterruptedly. They assimilate their food regularly, while in our climate, people diet on a little of everything, as the essayist has said, and our digestion and assimilation are frequently impaired. The liver becomes inactive and we do not assimilate our food as we should; consequently we are deprived of a great deal of the nutrition that should go to build up the hard tissues of the body.

As for the practical application of diet to the every day practice of dentistry, I question whether it is possible to control the diet of our patients to any great extent.

I have noticed in a number of cases, and one especially, where I have care of a physician's family in which there are two young men, one of whom is a great oatmeal eater, while the other does not eat any, that the one who does eat oatmeal has excessive decay, while the other who does not has scarcely any.

According to Prof. Miller's theory of dental decay, it seems to me that no matter how strong the teeth may be, they are as apt to decay under proper conditions as though they were soft. Probably not as rapidly, but they will decay just the same.

I presume the theory is well known to all of us, that the fermentation of glucose between the teeth forms the bi-product lactic acid, which dissolves out the lime salts of the teeth. No matter what the diet may be this process goes on regardless.

A word concerning mastication may not be amiss.

The process of mastication is a cleansing one, and also invigorating so far as the teeth are concerned. It is the first law of nature to remove whatever we have no use for, which undoubtedly accounts for the condition found in the epicurean classes.

As to the weapons given us with which to combat decay, the first and most important is cleanliness. Lime water has done its duty and been relegated to the past, giving place to the more modern, pleasant and effective milk of magnesia, which I have used extensively with very gratifying results to myself and my clientage.

In closing, if you will allow me to digress from the subject, I would like to mention the use of tonics, with people of low vitality and those who are nervous and irritable, thus permitting many operations that would otherwise be impossible.

Dr. Waugh: The ground has been so thoroughly covered by the essayist that there is but little left to say, but I would like to mention a little of my experience, as to the equability of climate and the effect it has upon the teeth during my travels of about four years in the South Sea Islands and south of the equator. Honolulu enjoys one of the most equable climates in the world. The food there consists almost entirely of a vegetable nature. I found in Honolulu people with the finest teeth I ever saw. I then went to the Samoa Islands. In that country I found them almost equal to the people of the Sandwich Islands. Then going to New Zealand, I found people with teeth equally as poor as our own. I traveled through New Zealand, Australia and Tasmania about two and a half years, and being a dentist, I of course paid particular attention to it, calling upon different dentists who were always courteous. Now, they are in an equable climate. I suppose there is a variance of about 42 degrees in the whole year. In South America, Buenos Ayres and Montevideo, they have just about the same grade of teeth that we have here; they eat and drink just about the same as we do. In Brazil I found people generally with elegant teeth. Now, I do not think that the change of climate has that deterious effect upon the teeth that the essayist states, that at least has not been my experience.

Dr. Holheinz : There is only one point I would mention to which my attention has been called. It seems to me that the people are consuming more and more year by year of prepared food. Go into a grocery store, and you will find the shelves piled high with green oats, wheat and corn, all ready to be used on the table and to be eaten with a spoon, which leaves no chance for mastication, very little chance for mixture with the saliva, and I venture to ask : If this continues to increase, what will the teeth in the future man be ?—*The Odontographic Journal.*

---

ARTICLE VI.

